# Modelling pyruvate dehydrogenase under hypoxia and its role in cancer metabolism

**DOI:** 10.1098/rsos.170360

**Published:** 2017-10-25

**Authors:** Filmon Eyassu, Claudio Angione

**Affiliations:** Department of Computer Science and Information Systems, Teesside University, Middlesbrough, UK

**Keywords:** constraint-based models, flux balance analysis, cancer metabolism, pyruvate dehydrogenase, hypoxia

## Abstract

Metabolism is the only biological system that can be fully modelled at genome scale. As a result, metabolic models have been increasingly used to study the molecular mechanisms of various diseases. Hypoxia, a low-oxygen tension, is a well-known characteristic of many cancer cells. Pyruvate dehydrogenase (PDH) controls the flux of metabolites between glycolysis and the tricarboxylic acid cycle and is a key enzyme in metabolic reprogramming in cancer metabolism. Here, we develop and manually curate a constraint-based metabolic model to investigate the mechanism of pyruvate dehydrogenase under hypoxia. Our results characterize the activity of pyruvate dehydrogenase and its decline during hypoxia. This results in lactate accumulation, consistent with recent hypoxia studies and a well-known feature in cancer metabolism. We apply machine-learning techniques on the flux datasets to identify reactions that drive these variations. We also identify distinct features on the structure of the variables and individual metabolic components in the switch from normoxia to hypoxia. Our results provide a framework for future studies by integrating multi-omics data to predict condition-specific metabolic phenotypes under hypoxia.

## 1. Introduction

In recent years, the use of metabolic models and their integration with multi-omics data has been extensive in better understanding the dynamics of disease processes [1]. Hypoxia, low-oxygen tension, is a feature of most tumours, which arise from uncontrolled proliferation of cancer cells. This microenvironment upregulates the formation of angiogenic factors initiating vascularization of the tumour cell [2]. Under this condition, the rate of glycolysis is enhanced, resulting in increased lactate production, while maintaining ATP levels and the biosynthesis of building blocks, including nucleotides and protein synthesis.

While metabolism has been seen merely as a marker for a long time, it is now widely accepted that it constitutes a driver of cancer; all cancer cells exhibit altered metabolism. One altered metabolic feature is an increased uptake of glucose through aerobic glycolysis, which is referred to as ‘Warburg effect’ [3,4]. In most cancer cells, downstream glycolytic enzymes and glucose transporters are overexpressed driven by hypoxia-inducible factor (HIF-1), along with oncogenes (such as PI3K, Ras, mTOR and Akt) and loss of tumour suppressors (PTEN and p53) [4–6]. In addition to over-simulating glycolytic enzymes, in rapidly growing tumours, HIF-1a regulates mitochondrial pyruvate dehydrogenase (PDH) leading to metabolic reprogramming. Inhibition of PDH by HIF-1a reduces mitochondrial respiration by reducing the flux of glucose to the tricarboxylic acid cycle (TCA cycle) [7].

PDH is an important enzyme that links and controls the flux between glycolysis and the TCA cycle. The PDH complex is a nuclear-encoded, mitochondrial matrix essential multi-enzyme component that catalyses the irreversible conversion of pyruvate to acetyl-CoA. It is a member of the α-ketoacid dehydrogenase complex family [8], which includes the α-ketoglutarate dehydrogenase complex (KGDHC) and the branched-chain α-ketoacid dehydrogenase complex (BCDHC). The PDH complex consists of three protein subunits: E1 (pyruvate dehydrogenase), E2, (dihydrolipoamide acetyltransferase; DLAT) and E3, (dihydrolipoamide dehydrogenase; DLD). The E1 enzyme consists of two alpha and two beta subunits; it is involved in removal of the carboxyl group from pyruvate and the transfer of the acetyl group to the E2 enzyme [9]. E3 is common to all three α-ketoacid dehydrogenases. These three subunits have distinct catalytic activity and depend on thiamine pyrophosphate (ThPP), lipoamide and FAD as cofactors to catalyse the overall reaction, respectively [8]. PDH is highly regulated through transcription factors and oncogenes in most cancers [4–7]. Cancer cells adopt their metabolism to achieve their energetics demand. PDH regulates this adaptation by controlling glucose metabolism and by linking glycolysis and the TCA cycle.

Metabolism is the only biological network that can be modelled reliably at a genome scale [10]. This enables systems-level understanding of the mechanisms of disease factors and provides a unique insight into the development of future therapies [11–14]. An increasing number of metabolic models (including genome-scale models) have been reconstructed for human metabolism [15–19]. Furthermore, the number of multi-omic integration methods to exploit such models is rapidly increasing [20,21]. Owing to the growing numbers and size of metabolic models, Bayesian, machine-learning and optimization techniques are being employed in this field to analyse and characterize metabolic features [10,22,23].

Given the critical role of PDH in controlling glucose flux between glycolysis and the TCA cycle and its role in metabolic reprogramming in cancer cells, we model and investigate the role of PDH using a blend of constraint-based modelling and machine-learning approaches. We develop a constraint-based model of cancer metabolism and present the action of PDH, characterizing metabolic phenotypes seen during hypoxia. We employ machine-learning methods to infer the reactions that contribute to the change in the metabotype from normoxia to hypoxia. To the best of our knowledge, this is the first study to use constraint-based modelling and machine-learning techniques to understand the metabolic role of PDH under hypoxia.

## 2. Material and Methods

### 2.1. Construction and curation of the metabolic model

To simulate PDH complex activity during hypoxia, a model of human metabolism was constructed based on the human metabolic model iAS253 [15]. The model was expanded based on interaction networks to include additional mitochondrial matrix reactions. The MitoMiner database [24] was used to find all proteins with evidence of mitochondrial localization. This information was supplemented with annotation from UniProt [25] and the Gene Ontology [26]. MitoMiner was then used to find KEGG reactions corresponding to each protein [27]. To confirm whether the new reactions are present in the mitochondrial matrix and whether reactions should be added to the expanded model, each reaction was manually examined by cross-referencing the information with BRENDA [28], HumanCyc [29] and relevant literature. We chose to use this model over genome-scale models such as Recon 2 [18] because small-scale models offer greater advantages for our application, particularly in enabling extensive manual curation, easier interpretation of biological data and visualization of flux distributions. The model is freely available in SBML format (see the electronic supplementary material, S1) for academic use, and can be used to study cancer metabolism as well as other human metabolic disorders.

### 2.2. Modelling pyruvate dehydrogenase using flux balance analysis

Flux balance analysis (FBA) is a widely used mathematical approach for modelling large-scale metabolic networks [30]. Because FBA assumes the homeostasis of a system (constant internal metabolite concentration *x*), it does not need knowledge of metabolite concentrations and enzyme kinetics. This differentiates FBA from other modelling techniques that require kinetics parameters, usually are difficult to obtain. In FBA, the set of biochemical reactions is represented mathematically in the form of a stoichiometric matrix *S*; constraints are placed on individual reactions to limit the allowable flux *ν* each reaction can carry. A unique solution is obtained by defining the objective function through a vector *c*; this is a measure of how each component in the network contributes to the production of a biologically desirable phenotype.

Formally, we adopt the following linear program:

$$\begin{array}{rl} & \max\;c\cdot v,\;\text{such that}\\ & S\cdot v=\dot{x}\\ & V\text{min}\leq v\leq V\text{max}\\ & {\dot{x}}_{i}=0\;if\;M_{i}\in \text{internal metabolites}\\ & {\dot{x}}_{i}\in R\;if\;M_{i}\in \text{exchange metabolites}. \end{array}$$

For a detailed description of FBA, we refer the reader to the work of Orth *et al.* [30]. To represent the major role of the mitochondrion, primarily energy production, we used maximum ATP production as the objective function. Linear programming was then used to calculate the optimal flux distribution that maximizes the objective function. The model was encoded in SBML format [31] and fulfils MIRIAM requirements [32]. Simulations were carried out in Matlab (version R2016a) using the COBRA toolbox [33] with the linear programming solver GLPK. All flux values were given in micromoles per minutes per grams of dry weight (μmol min^−1^ gDW^−1^).

To represent hypoxia, the maximum allowable uptake flux of oxygen was reduced to 0 µmol min^−1^ gDW^−1^ in intervals of 10% from its level under normal conditions (19.8 μmol min^−1^ gDW^−1^). The uptake value of 10% oxygen was chosen to reflect the typical conditions used in experimental hypoxia studies [34–43]. For the first simulation, only the availability of oxygen was reduced while the uptakes of metabolites into the system were kept unchanged [15]. Flux balance analysis was used to determine effect of PDH and rate of ATP production during hypoxia.

To determine the effect of PDH on ATP production during hypoxia, the activity of PDH was reduced from its maximum flux capacity to zero. It is well established that the concentration of some metabolites is elevated during hypoxia in cancer cells. To further evaluate this, simulations were run where the uptake of amino acids were unconstrained by increasing the upper uptake bound.

## 3. Results and discussion

A manually curated human metabolic model was created to model and investigate the metabolic effects of PDH during hypoxia (see Material and methods). The model is composed of 616 reactions, 765 metabolites, 90 boundary reactions and 92 transport steps (electronic supplementary material, S1). These transport steps represent the human mitochondria carrier family [44] and are modelled to reflect the physiological role of each transport mechanism. Given the complexity of the role of PDH in cancer metabolism, our model offers better advantages in interpretation of biological data and easier visualization of flux data over genome-scale models such as Recon 2.2 (table 1).

**Table 1.** Comparison between ***IFEAS616*** and Recon 2.

	*iFEAS616*	Recon 2.2 [45]
reactions	826	7785
metabolites	765	5324
genes	332	1675

### 3.1 Pyruvate dehydrogenase mechanism under hypoxia

**Figure 1.**
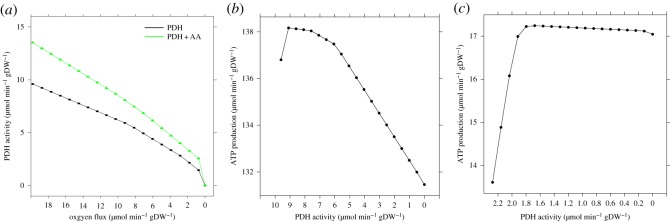
Effect of hypoxia on pyruvate dehydrogenase (PDH) and ATP production. (*a*) The flux of PDH was determined, when oxygen availability was reduced from 100% (normoxia) to 0% (anoxia) of its normal value. To represent PDH effect on metabolism seen in cancer cells, uptake of amino acid was unconstrained (PDH + AA). In all conditions, the rate of PDH gradually decreased as the availability of oxygen decreased. (*b*) The rate of ATP production was determined by varying the flux of PDH from its maximum flux, while oxygen levels were kept constant at 100%. A maximum ATP production flux of 138.1 µmol min^−1^ gDW^−1^ was reached. For levels of PDH flux lower than 9 µmol min^−1^ gDW^−1^, the ATP level declined regularly, with an increasing negative slope. (*c*) Under hypoxia, oxygen availability was reduced to 10% of its normal value. ATP production increased above 17.2 µmol min^−1^ gDW^−1^, but for activity of PDH lower than 1.8 µmol min^−1^ gDW^−1^, it decreased to 17 µmol min^−1^ gDW^−1^. Interestingly, the decay observed in hypoxia is slower than in normoxia conditions.

**Figure 2.**
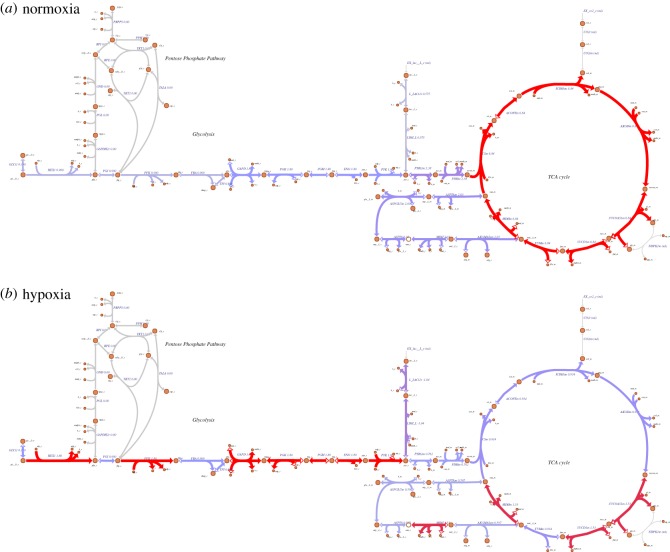
Metabolic map depicting glycolysis, the pentose phosphate pathway and TCA cycle during (*a*) normoxia and (*b*) hypoxia. The flux distribution of central metabolism from simulation of normoxia and hypoxia is shown, where glycolysis starts from the uptake of glucose from the extracellular compartment and its transport into the cytosol by glucose transporter. In the cytoplasm, glucose is metabolized and converted to pyruvate and fed to the TCA cycle during normoxia. However, under hypoxia, the majority of glucose is converted to lactate and exported to the extracellular compartment by lactate transporter, which in turn maintains the redox potential. The flux distribution under both conditions is illustrated. We used Escher [46] to generate the metabolic map, flux values for each reaction are indicated. Line thickness and colour (grey to red) corresponds to the flux value. Negative flux values indicate the corresponding reaction is operating in the reverse direction.

To determine PDH action under hypoxia, the maximum allowable uptake flux of oxygen was reduced from normoxia (19.8 µmol min^−1^ gDW^−1^) to hypoxia (1.9 µmol min^−1^ gDW^−1^) in intervals of 10%. The flux output of PDH gradually declined as the rate of oxygen availability was reduced from normoxia to anoxia (figure 1*a*). Under these simulations, lactate and succinate accumulated at a rate of 1.04 and 0.59 µmol min^−1^ gDW^−1^, respectively (figure 2; electronic supplementary material, S2) consistent with reports of hypoxia studies in the literature [34–43]. Succinate accumulated entirely from the TCA cycle as an end-product of central metabolism, resulted from reduced capacity of the oxidative phosphorylation (OXPHOS) to metabolize succinate.

Under aerobic respiration, succinate and electron-rich metabolites (such as NADH generated from the TCA cycle and fatty acid oxidation) are oxidized by complexes II and I of the electron transport chain, respectively. Oxidation of succinate (an intermediate of the TCA cycle) by complex II transfers electrons to ubiquinone, a small lipid-soluble molecule that can be reduced and oxidized, and which freely diffuses within and across the inner mitochondrial membrane. The reduced ubiquinol (UQH_2_) diffuses to complex III where it is reoxidized; at the same time, complex III reduces cytochrome *c*, which in turn reduces complex IV, where oxygen is used as terminal electron acceptor and reduced to water. The energy from this chain of electron transfers is used by the respiratory complexes to transport protons across the mitochondrial membrane, so generating the proton motive force and the electrochemical potential across the mitochondrial membrane that is used by mitochondrial ATP synthase to form ATP from ADP and phosphate. Under hypoxia, this mechanism diminishes owing to the lack of oxygen availability. Therefore, the rate of succinate oxidation by OXPHOS is reduced, leading to its accumulation.

In our model, as expected, the majority of glycolytic-derived pyruvate was converted to lactate (figure 2). The production of lactate allowed the removal of NADH generated from glycolysis owing to the impaired capacity of the OXPHOS to oxidize NADH efficiently. This is consistent with the well-known phenomena seen in cancer cells, namely that under hypoxia glycolytic rate is often enhanced leading to increased production and accumulation of lactate [47]. Through this mechanism, glycolysis maintains ATP levels and provides building blocks for nucleotide and protein synthesis, which is essential in cancer cells to maintain proliferation through many transcription factors and oncogenes [48,49].

Consistent with our findings, hypoxia directly increases lactate production and accumulation owing to the effect of the changes in the mitochondrial redox status caused by reduced oxygen availability. Under hypoxia, the NAD reduced : oxidized (NADH : NAD+) ratio in the mitochondria is high owing to the reduced capacity of the electron transport chain and consequent reduction in the rate of NADH oxidation by complex I [2]. Subsequently, this reduces NADH-producing reactions in the TCA cycle and oxidation of fatty acids, thereby reducing the transfer of glycolytic-derived NADH by the malate-aspartate shuttle into the mitochondrial matrix. Thus, the production of lactate and its accumulation is energetically favourable.

The production of lactate by lactate dehydrogenase (LDH), which leads to cellular acidification is a contributing factor for the malignancy of cancer cells [50]. LDH consists of two subunits (LDHA and LDHB); upregulation of LDHA through HIF1 results in pyruvate reduction in hypoxia [51,52]. In addition, HIF1 has been shown to upregulate almost all glycolytic genes encoding enzymes and glucose transporters including hexokinase 1 and 3, aldolase A and C, and glyceraldehyde 3-phosphate dehydrogenase, leading to metabolic reprogramming of glycolysis [51–54].

Furthermore, pyruvate dehydrogenase kinase 1 (PDK1) inhibits PDH during hypoxia leading to reduced mitochondrial respiration and reactive oxygen species (ROS) production [55]. PDK1 phosphorylates and inhibits the PDH complex, reducing the conversion of pyruvate to acetyl-CoA, thereby regulating the flux of metabolite to the TCA cycle. Our simulations, under both active and inactive PDK1 activity during hypoxia (and normoxia) showed no change on the mechanism of PDH, which should be considered an incorrect prediction of the model when maximum ATP production is used as an objective function. This suggests that phosphorylation events can be introduced through metabolic constraints, directly on the flux rates (e.g. through decreasing PDH flux as in figure 1) or through flux bounds, e.g. shrinking upper and lower limits to simulate inhibition. Another possible approach could involve modifications of gene–protein–reaction rules of the PDH reaction to include PDK1, although this would require the adoption of multi-omic extensions of FBA to simulate variable PDK1 activity levels.

Finally, further simulations were performed to determine whether additional sources of metabolites enhanced PDH capacity. The uptake rate of amino acid metabolites in the model was unconstrained. This enhanced PDH capacity by 29%, but as the levels of oxygen fell, PDH action decreased steadily (figure 1*a*).

### 3.2 Pyruvate dehydrogenase action on ATP production

To determine whether PDH affects the rate of ATP production under normoxia and hypoxia, we quantified ATP production while varying PDH in 10% decrement of its maximal flux. Figure 1*b* shows a gradual decrease in ATP production when PDH flux was decreased. More specifically, the decrease of PDH activity caused ATP production to decline only by 5% (from 138 to 131 µmol min^−1^ gDW^−1^). This suggests that the majority of the source of ATP under normoxia is driven by alternative fuel sources, including fatty acids. Under normal levels of oxygen availability, ATP production through OXPHOS was primarily generated from β-oxidation of fatty acids, which generates energy-rich NADH and acetyl-CoA. Acetyl-CoA subsequently enters the TCA cycle generating further electrons and succinate. The efficient capacity of the electron transport chain then oxidizes NADH and succinate to generate ATP as described above.

Under hypoxia (figure 1*c*), when oxygen was further reduced, the contribution of OXPHOS to ATP production was gradually diminished. This was owing to the electron transport chain capacity to use oxygen being restricted and its inability to use fatty acid as an energy source. Reduced capacity of electron transport chain inhibits TCA cycle and NADH-producing reactions (including PDH) in the mitochondrial matrix, such as fatty acid oxidation. Therefore, under this condition, the action of PDH is reduced by 78% compared to conditions under normal oxygen supply, subsequently reducing the rate of ATP production (figure 1*b*,*c*).

Under hypoxia, alternative carbon sources are used—amino acids, e.g. glutamine, lysine, proline and valine. The mechanism in which glutamine (as a fuel source) enters central metabolism is as follows: glutamine is converted in a two-step reaction to glutamate and then oxoglutarate by hydrolysing glutamine to glutamate and ammonia (which accumulated) by glutaminase. Glutamate was imported directly into the mitochondrial matrix and further converted to oxoglutarate and ammonia by glutamate dehydrogenase. In the TCA cycle, oxoglutarate was converted to succinyl-CoA and then succinate, subsequently used for ATP production. Consistent with this, under hypoxia, glutamine metabolism maintains ATP synthesis through OXPHOS in tumour cells [56–58]. In addition, glutamate in cancer cells activates reductive carboxylation through isocitrate dehydrogenase [59] and production of mitochondrial NADPH through the activity of malic enzyme, a mitochondrial reaction which converts malate to pyruvate, and is found to be expressed at high levels in some tumours [57].

### 3.3 Principal component analysis reveals hypoxia-versus-normoxia biomarkers

Machine learning in biology can be used to reveal unique characteristics from multidimensional and high-throughput data [60,61]. Two broad types of methods exist in machine learning: supervised and unsupervised learning. Unsupervised learning methods such as principal component analysis (PCA) identify feature similarities in datasets. PCA is an eigenvector-based analysis that quantifies directions that best explain variance in the data. In our case, it is based on the singular value decomposition (SVD) of the flux rates, and is therefore equivalent to finding the system of axes in the space of flux rates such that the covariance matrix is diagonal. PCA defines new variables, called principal components, as linear combinations of the existing variables.

**Figure 3.**
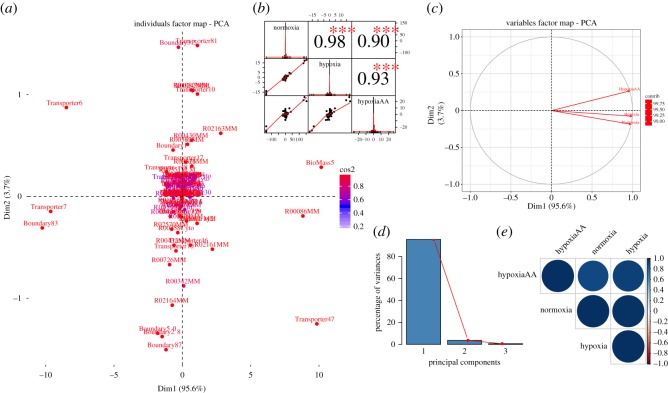
Principal component analysis of flux rates. (*a*) Individual factor map. Biomass5 (ATP production) and some reaction of OXPHOS (i.e. R00086MM and R02163MM) are distinguishable between the different conditions, and therefore constitute the main metabolic biomarkers of hypoxia. (*b*) Correlation histogram. Top panel shows significance level (*p*-value < 0.001) associated with the corresponding correlation coefficients. Diagonal panel shows the distribution of each variable. (*c*) Variables factor map. The first component (Dim1) correlates almost perfectly with the variable hypoxia, while the second component correlates mostly with HypoxiaAA. (*d*) Scree plot generated from eigenvalue versus component number. (*e*) Correlation matrix illustrating the correlation between each variable. Positive correlation is displayed by a blue colour, while the colour intensity and size of the circles are proportional to the correlation coefficients. The reader is referred to the electronic supplementary material, S1 for individual reaction names, and to the main text for further details and interpretations.

We performed PCA on the aggregate flux dataset from normoxia, hypoxia and hypoxia with excess amino acid (HypoxiaAA) for all active reactions. Our goal was to identify key reactions that drive variation and investigate how these drivers change over different levels of oxygen availability (table 2 and figure 3*a–e*). The principal components always represent an orthogonal basis of the space of flux rates.

**Table 2.** Contribution of variables and individual reactions on the principal components. (The variable hypoxia is highly correlated with the first component (PC1), compared to variable HypoxiaAA. This variation is driven by excess amino acids present in HypoxiaAA in addition to the lack of oxygen. The individual reactions on the first two principal components show oxygen transporter (reaction id: Transporter47) and to some extent ATP production (reaction id: BioMass5) scored highly, indicating a significant effect on ATP synthase activity (see text for a more detailed description).)

condition		PC 1	PC 2	PC 3
	(*a*)			
normoxia		0.979	−0.185	0.083
hypoxia		0.991	−0.076	−0.108
HypoxiaAA		0.963	0.267	0.027
reaction ID	reaction description	PC 1	PC 2	PC 3
	(*b*)			
Transporter47	oxygen transporter	16.486	6.859	1.170
BioMass5	ATP production	17.570	0.360	0.259
R00086MM	ATP synthase	13.241	0.162	1.401
R02161MM	complex III	0.823	1.175	0.132
R02163MM	complex I	1.343	1.685	0.653
Boundary2	oxygen uptake	0.198	0.305	0.030

Using PCA on our flux dataset showed that the first two eigenvectors sum to more than 98% of the variance in the dataset (figure 3*a,b*). This indicates that changes in reaction fluxes are correlated under various levels of oxygen, and the changes following the uptake of additional amino acids are also correlated (table 2*a*). Plotting the first two singular vectors of PCA on the fluxes shows distinct features on the structure of the variable and individual components (figure 3*a,c*). We found that the first component (figure 3*d*, Dim1) correlates highly with variable hypoxia, while the second component correlates with the variable HypoxiaAA. The variation in each condition is driven by changes in addition to the amount of oxygen but also by the excess amino acids present in HypoxiaAA (table 2*a*). Addition of metabolites such as glutamine and arginine increased the rate of ATP production during hypoxia, by enhancing the availability of substrates (and electrons) required for energy generation. Figure 3*a* and table 2*b* show the individuals factor map plot of the principal component scores for individual reactions on the first two principal components. Superimposed on the plot are mean scores on the components for qualitative variables that are included in the PCA. The reactions ATP synthase (reaction id: R00086MM) and HypoxiAA ATP production (reaction id: BioMass5) scored highly with the first component, therefore indicating a large impact of ATP synthase activity in determining the normoxic or hypoxic phenotype.

Our results are in keeping with the fact that ATP synthase is highly dependent on the rate of oxygen and metabolite availability for generating ATP. In fact, the mechanism by which ATP is generated is as follows and depends on molecular oxygen under normal conditions. Oxidation of glucose begins with glycolysis in the cytoplasm generating NADH, ATP and pyruvate. Pyruvate is then transported into the mitochondrial matrix and is converted to acetyl-CoA and NADH by PDH. Subsequently, acetyl-CoA enters the TCA cycle and is oxidized to CO_2_ and water, yielding NADH and GTP. Electron-rich metabolites such as NADH generated from these pathways and oxidation of fatty acids, together with succinate HypoxiaAA are then oxidized by complexes I and II, respectively. As described above, the transfer of these electrons generates the electrochemical gradient across the mitochondrial membrane, which in turn is used by ATP synthase to form ATP.

## 4. Conclusion

The PDH complex regulates the metabolic adaptation by controlling glucose metabolism, used by cancer cells to meet the bioenergetics demands. To investigate the complexity of role of PDH in cancer metabolism, we used a small-scale constraint-based model; the choice of our model over genome-scale model was appropriate for in-depth interpretation of biological results.

Our results showed PDH action diminished during hypoxia. We identified metabolic markers of cancer metabolism (lactate production and its accumulation) in agreement with experimental findings in the literature. Taken together, the combination of our model, machine learning results and known biochemistry suggests that oxygen transporters and ATP synthase and production constitute clear biomarkers of hypoxic behaviour.

We envisage that our model will be useful to researchers for studying human diseases involving a hypoxia-like metabotype, including cancer. To expand and further investigate such diseases, the model can be used as a scaffold to integrate multi-omics data [61,62], and to predict condition-specific metabolic configurations, e.g. by dynamically changing flux bounds using transcriptomic and proteomic data.

## Supplementary Material

Model

## Supplementary Material

Flux distribution results

## Supplementary Material

Source code

## Data accessibility

The datasets supporting this article have been uploaded as part of the electronic supplementary material.
